# Genetic mutation of *Tas2r104/Tas2r105/Tas2r114* cluster leads to a loss of taste perception to denatonium benzoate and cucurbitacin B

**DOI:** 10.1002/ame2.12357

**Published:** 2023-12-28

**Authors:** Bowen Niu, Lingling Liu, Qian Gao, Meng‐Min Zhu, Lixiang Chen, Xiu‐Hua Peng, Boying Qin, Xiaohui Zhou, Feng Li

**Affiliations:** ^1^ Department of Laboratory Animal Science, Shanghai Public Health Clinical Center Fudan University Shanghai China; ^2^ Department of Biology, College of Life Sciences Shanghai Normal University Shanghai People's Republic of China

**Keywords:** bitter taste receptor, CRISPR/Cas9, genetic mutation, two‐bottle preference test, type 2 taste receptors (*Tas2rs*)

## Abstract

**Background:**

Bitter taste receptors (*Tas2rs*) are generally considered to sense various bitter compounds to escape the intake of toxic substances. Bitter taste receptors have been found to widely express in extraoral tissues and have important physiological functions outside the gustatory system in vivo.

**Methods:**

To investigate the physiological functions of the bitter taste receptor cluster *Tas2r106*/*Tas2r104*/*Tas2r105*/*Tas2r114* in lingual and extraoral tissues, multiple *Tas2rs* mutant mice and *Gnat3* were produced using CRISPR/Cas9 gene‐editing technique. A mixture containing Cas9 and sgRNA mRNAs for *Tas2rs* and *Gnat3* gene was microinjected into the cytoplasm of the zygotes. Then, T7EN1 assays and sequencing were used to screen genetic mutation at the target sites in founder mice. Quantitative real‐time polymerase chain reaction (qRT‐PCR) and immunostaining were used to study the expression level of taste signaling cascade and bitter taste receptor in taste buds. Perception to taste substance was also studied using two‐bottle preference tests.

**Results:**

We successfully produced several *Tas2rs* and *Gnat3* mutant mice using the CRISPR/Cas9 technique. Immunostaining results showed that the expression of GNAT3 and PLCB2 was not altered in *Tas2rs* mutant mice. But qRT‐PCR results revealed the changed expression profile of *mTas2rs* gene in taste buds of these mutant mice. With two‐bottle preference tests, these mutant mice eliminate responses to cycloheximide due to genetic mutation of *Tas2r105*. In addition, these mutant mice showed a loss of taste perception to quinine dihydrochloride, denatonium benzoate, and cucurbitacin B (CuB). *Gnat3*‐mediated taste receptor and its signal pathway contribute to CuB perception.

**Conclusions:**

These findings implied that these mutant mice would be a valuable means to understand the biological functions of TAS2Rs in extraoral tissues and investigate bitter compound–induced responses mediated by these TAS2Rs in many extraoral tissues.

## Graphical abstract

Multiple *Tas2rs* mutant mice and *Gnat3* were generated by Crispr/Cas9 gene‐editing technique. QPCR and immunofluorescence assay was employed to study the expression of bitter receptor in taste bud from these mutant mice. Furthermore, two‐bottle preference test revealed that these mutant mice showed a loss of taste perception to Quinine (QHCI), Denatonium Benzoate (DB), and Cucurbitacin B (CuB). *Gnat3*‐mediated taste receptor and its signal pathway contributes to CuB perception. These mutant mice would be a valuable means to understand the biological functions of TAS2Rs in extraoral tissues.

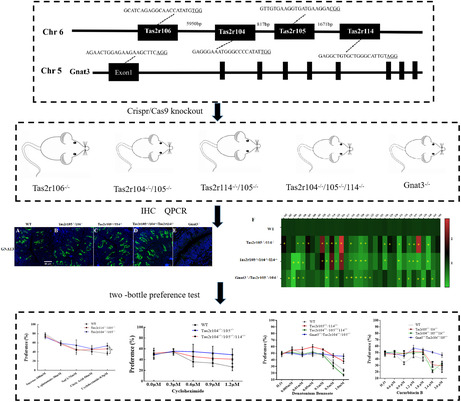

## 1 INTRODUCTION

Bitter taste receptors, also regarded as type 2 taste receptors (*Tas2rs*), belong to the superfamily of G‐protein‐coupled receptors (GPCRs) and are generally considered to sense various bitter compounds and help animals escape the intake of toxic substances.^1^ Bitter taste receptors have been found to be widely expressed outside the gustatory system.^2^, ^3^, ^4^, ^5^ Several studies with *Tas2r143*‐CreERT2 transgenic mouse,^6^ *Tas2r105*‐Cre/GFP transgenic mouse,^7^, ^8^, ^9^ and *Tas2r131*‐Cre knock‐in mouse^10^, ^11^ showed that bitter taste receptors were expressed outside the gustatory system. Such solitary chemosensory cells are capable of sensing bacterial quorum sensing molecules in the respiratory tract^12^, ^13^, ^14^ and can induce type 2 immune responses to helminth infections in the murine intestinal tract.^4^ Furthermore, susceptibility to upper respiratory tract infection is associated with *TAS2R38* polymorphisms in humans.^15^, ^16^ Taste receptor polymorphisms are even linked to male infertility by altering sperm cilia motility and downregulating meiotic cell division.^3^ This indicates that bitter taste receptors have important physiological functions in extraoral tissues in vivo.

Humans and mice have 25 and 35 functional bitter taste receptors, respectively.^17^, ^18^, ^19^ Muroid cluster I contains *Tas2r107*, *Tas2r106*, *Tas2r105*, *Tas2r114*, and *Tas2r104*, which have a common ancestor with human TAS2R10.^17^, ^18^ *Tas2r104*, *Tas2r105*, and *Tas2r114* are organized as a head‐to‐tail array within a single 6‐kb DNA fragment. This may be regarded as a representative of a single human gene being orthologous to multiple mouse genes, that is, one‐to‐multiple orthology.^20^ Our study using bacterial artificial chromosome transgenic mice has revealed the different expression patterns of muroid cluster I in vivo.^7^, ^8^, ^9^, ^21^ Low to moderate expression levels of murine *Tas2rs* were found in extraoral tissues such as kidney, gut, testis, and heart.^2^, ^21^, ^22^, ^23^, ^24^ In particular, *Tas2r114* with the lowest expression in lingual papillae^25^ showed moderate to high expression in kidney,^21^ gut,^21^ and testis.^24^ Heterologous expression analysis of *Tas2rs* in HEK293T cells showed that these *Tas2rs* were responsive to different bitter compounds.^25^ Therefore, it is tempting to speculate that these *Tas2rs* in muroid cluster I may discern a different set of the bitter compounds and have different physiological functions in vivo. To investigate the physiological functions of bitter taste receptor cluster *Tas2r106/Tas2r104/Tas2r105/Tas2r114* in gustatory and extraoral tissues, multiple *Tas2rs* knockout (KO) mice were produced using CRISPR/Cas9 gene‐editing technique. Using two‐bottle preference tests, we observed that these *Tas2rs* mutant mice have a decreased response to the cycloheximide (CYX), quinine dihydrochloride (QHCl), denatonium benzoate (DB), and cucurbitacin B (CuB).

## 2 MATERIALS AND METHODS

### 2.1 Animals

This study was approved by the Animal Care and Use Committees of Shanghai Public Health Clinical Center (GW2018‐A055‐01, 2023‐A030‐01). Animals were kept and reproduced in a specific pathogen‐free animal facility at the Department of Laboratory Animal Science, Shanghai Public Health Clinical Center (five mice per cage), with free access to food and water.

### 2.2 sgRNA and Cas9 mRNA in vitro transcription

The MIT CRISPR Design Tool was employed to analyze target sites at the *Tas2rs* gene and *Gnat3*.^26^ gRNAs with the highest scores were chosen. A ligation‐free PCR approach for sgRNA template synthesis was used in this paper.^27^ A 60‐bp target‐specific oligo, including the T7 promoter, was synthesized, and the sgRNA template was generated using PCR with several sets of universal primers. The primers used are presented in Table 1. In vitro transcription and RNA purification were performed using the conventional methods, as previously described.^27^ Final products were stored at −80°C until further analysis.

**TABLE 1:** Oligonucleotides for genotyping, surveyor assay and IVT‐1 in this study.

Gene	Forward primer	Reverse primer
*Primer for qRT‐PCR*		
*mTas2r102*	GGAAGCTTGGTGTTCTTGCTTGG	AGATCAGCTCGGTCCACATTGC
*mTas2r103*	ATTAGCACTGGGTTTACACTCACC	CCACAGGGAGAAGATGAGCAGAAG
*mTas2r104*	CCTTCTGACTATGCTTATGC	AGGATGTGTTGCTCTGATAT
*mTas2r105*	CAGAGACCTCAACACAGAA	ATGAGTGACAGCAAGGATAT
*mTas2r106*	CTCACAGGCTTGGCTATT	CAGGAGATAGAAGAGGTTGA
*mTas2r107*	TCCCTGCGGTCACTCAATCATC	CAGTGCCTTCAAAGAGGCTTGC
*mTas2r108*	ACAGTCGCAGAATTGCCTCTCC	AGGAATCTAGTGATGGCCAAGCTG
*mTas2r109*	AAGGAAGAAACCTCCTTTCGTTGC	AGAGAGGCATGTCTCAGCTTTCTG
*mTas2r110*	AGGTCAATGCCAAACCACCT	ACTGGCTTGTCTCAGCTTGT
*mTas2r113*	TCCGCACTGCTCTGGCAATTAG	TGAACAGACACCCACCAATCTAGG
*mTas2r114*	GCTGTCTCCTGTCAACATAA	AACCATCTTCGCAACAACT
*mTas2r115*	AGAGAAGAACGTTCCCTTCAGCAG	ACCCATATCAGAGCAAGCCTGGAG
*mTas2r116*	AACACAGTGCCCATGGATGCAG	ATGTCTGCAAGGCTCTGATGTGG
*mTas2r117*	CACTGTTGGTGTCATTGCTCC	GGACAAAACAACGGGGACAG
*mTas2r118*	AAGTTGCACAACGGTTGCAGTG	TCTCCACCGGTGACAGTCTTTG
*mTas2r119*	TCACACCCACAAGAAGGAGCAC	ACCTTAAGGATGGAGAACCTGCAC
*mTas2r120*	TCCTTCTACCCAGCAGGTCATTC	AGCATCTCATCTGCCTCAGCAAC
*mTas2r121*	GCTTGAAGGGTAGAAAAGCCC	GAACGAGACCCCAGCACTAAA
*mTas2r123*	TGCAGGTCAATGCCAAACAACC	CAGCAGGAAGGAGAACACAGTTTG
*mTas2r124*	AGTCTCTGGCTTGCTACAGCTC	AGCTTCCCAGAAGCATGTGGAC
*mTas2r125*	ATCTTCTCCCTGTGGAGACACCTG	TGGTGTCTTCGGAGCCTTTAGC
*mTas2r126*	CCCGGCAGCTCATTAGTCTT	CGGACACCAAGATAGAGCCC
*mTas2r129*	TTGCAGATGCCCACATCAGAGTC	TGGCACAGAGTAGGACATAGGTG
*mTas2r130*	GACAGAGGCATGTCCAGCTT	CCACCTGCCTCAGCATTTTC
*mTas2r131*	ATCAACATGGCTTGCCACCTG	AGCACACCTCTCAATCTCCACTTC
*mTas2r134*	GCTGTCATGCGATGCTGATG	GTGAGCCTGGGTGCTGTAAT
*mTas2r135*	TCAGTTCTGCCAGCAACACACC	TGAATCACCACCTGCCACATCC
*mTas2r137*	AGCATACATTTGTGGCCATGCTC	AAGCAGAGGGTCCCTTAGATCCAG
*mTas2r138*	TGCTATTCAGCTCGCCTGCTTC	TGGCTTGGTAGTTGTGGCTCAG
*mTas2r139*	GCTTCTGTGGCTCTCAGTGT	AGAGTAGCTGTTGCGACGAA
*mTas2r140*	CATGCAACACAATGCCAAAGACTC	AGGGCCTTAATATGGGCTGTGG
*mTas2r143*	TTCCCAGGCTGCTGGTTGTATC	AGTTCCCGGTGGCTGAAATGAC
*mTas2r144*	CACGTGGGTGCCATCAAATC	TGAACATGGTGCTGAAACCG
*mGapdh*	TGGCCTTCCGTGTTCCTAC	GAGTTGCTGTTGAAGTCGCA

Gene	Forward primer	Backward primer	Size (bp)
*Primers for genotyping and amplifying the sgRNA targeted fragment*			
*Tas2r105*	ATGGATGACTGTGAATCACCTG	CATGAGCTTCTGTGTTGAGGTC	410
*Tas2r104*	CTTCTTGGAAACTGGCAGAGAT	TTCATGTGAGCTTCTGTGTTGA	697
*Tas2r114*	TGCTGAGCACAATGGAAGG	GTGGCAAACCAGACATTCAA	301
*Tas2r106*	AAAAGCCGTTACTGGGTCAC	GTCCTTGTCTTTCTTATGGC	1069
*Tas2r104/105*	CAGGGAAAGTGGACAGAACTC	CTGCTGAACTGTGCCAGAAC	2843

Name	Primer sequence
*Primer to produce the template for sgRNA and Cas9 in vitro transcription*	
T7‐Cas9‐Fwd	TAATACGACTCACTATAGGGAGATTTCAGGTTGGACCGGTG
Cas9‐Rev	CTATTAATAACTAGCGGCCG
Scaffold‐Fwd	GTTTTAGAGCTAGAAATAGC
Scaffold‐Rev	AAAAGCACCGACTCGGTGCC
T7‐Fwd	GAAATTAATACGACTCACTATAGGGAGA
T2R114‐gRNA	TACGACTCACTATAGGGAGAGAGGCTGTGCTGGGCATTGTGTTTTAGAGCTAGAAATA
Tas2r106‐gRNA	TACGACTCACTATAGGGAGAGCATCAGAGGCAACCATATGGTTTTAGAGCTAGAAATA
Tas2r105‐gRNA	TACGACTCACTATAGGGAGAGTTGTGAAGGTGATGAAGGA GTTTTAGAGCTAGAAATA
Tas2r104‐gRNA	TACGACTCACTATAGGGAGAGAGGGAAATGGGCCCCATATGTTTTAGAGCTAGAAATA

Abbreviation: qRT‐PCR, quantitative real‐time polymerase chain reaction.

### 2.3 Cas9/sgRNA coinjection of one‐cell embryos

Female C57BL/6J mice (aged 4–6 weeks) were superovulated and then mated to produce the fertilized embryos. The Cas9/sgRNA coinjection method was used, as previously described.^28^ Microinjection was performed using a Leica DMi8 inverted microscope with the Eppendorf microinjection system (FemtoJet 4i/FemtoJet 4×). The Cas9/sgRNA mixture (50 ng/μL of sgRNA and 100 ng/μL of Cas9 mRNA) was microinjected into the cytoplasm of the zygotes. Two‐cell embryos were transferred into the oviduct of pseudopregnant Institute of Cancer Research female mice.

### 2.4 T7EN1 cleavage and sequencing

Mouse genomic DNA was extracted from mouse tail tips using routine methods, as described previously.^28^ Hieff Canace high‐fidelity DNA polymerase (YEASEN, 10135ES80) was used to amplify target sites. Primers are presented in Table 1. Then, T7 endonuclease I (T7EI, M0302L, NEB) was used to digest hybridized PCR products, as described previously.^28^ Target region amplicons from mutant founders were cloned into a pESI‐Blunt vector (YEASEN, 10909ES20) and sequenced by Sangon Biotech. Positive F0 founders were crossed with C57BL/6J mice to screen whether mutation was transferred to the next generation.

### 2.5 RNA extraction of circumvallate taste buds, reverse‐transcription PCR, and quantitative real‐time PCR

The procedure to prepare the circumvallate papillae closely followed published protocols.^29^, ^30^ Briefly, the tongue was immediately removed and placed in ice‐cold Tyrode's solution after mice were killed. An enzyme cocktail (1 mg of collagenase A [Roche Diagnostics, Monza, Italy], 2.5 mg of dispase II [Roche Diagnostics], and 1 mg of trypsin inhibitor type I‐S [Sigma] in 1 mL of Tyrode's solution) was then injected between the lingual epithelium and the muscle layers. After incubation in divalent‐free Tyrode's solution at room temperature (20°–22°) for 30–40 min with air bubbling, the lingual epithelium was carefully peeled free from the underlying tissue. Finally, circumvallate papillae were softly dissected under a dissecting microscope and immersed in ice‐cold Tri‐Reagent (Trans, Beijing). Adjacent epithelium without fungiform taste buds was used as control.

Total RNA was extracted from taste buds using Tri‐Reagent (Trans) following the manufacturer's protocol. DNAaseI (Roche) was used to eliminate the residual genome; 1 μg of isolated RNAs from each sample was reverse‐transcribed into complementary DNA (cDNA) using the Hifair II 1st Strand cDNA Synthesis Kit (Yeasen Biotech).

Quantitative real‐time polymerase chain reaction (qRT‐PCR) was carried out to investigate the expression levels of *Tas2rs* using Hieff UNICON Universal Blue qPCR SYBR Green Master Mix (Yeasen Biotech) following the manufacturer's protocols; 10 ng of cDNA was added in each tube. The cycling conditions for qRT‐PCR were 95°C for 10 min, followed by 40 cycles of 95°C for 10 s, 60°C for 30 s, and 72°C for 1 s. The fold increase in mRNA expression was calculated using the routine 2^−ΔΔ*Ct*^ method.^31^, ^32^, ^33^ All the primers for mouse *Tas2rs*, *Gnat3* are presented in Table 1 and have been used in our previous study.^7^, ^9^

### 2.6 Immunostaining

Routine methods were used in immunostaining for taste buds, as described previously.^34^, ^35^ Briefly, the tongue was dissected, postfixed in 4% neutral paraformaldehyde for 12 h, and placed in 30% sucrose at 4°C overnight; 10–12 μm cryosections were cut and affixed to glass slides. Staining against polyclonal anti‐GNAT3 (sc‐395, Santa Cruz Biotechnology) and anti‐PLCβ2 (sc‐22458, Santa Cruz Biotechnology) was performed using the standard immunofluorescence procedure.^31^, ^32^, ^36^ Blocking peptide for anti‐GNAT3 (sc‐395P, Santa Cruz Biotechnology) was used to specifically block GNAT3 antibody. After the cryosections were washed in phosphate‐buffered saline (PBS, 3 × 10 min), they were kept in blocking buffer for 1–2 h and then incubated for 36–48 h at 4°C in the polyclonal antibody: anti‐PLCB2 (1:500), anti‐GNAT3 (1:500), or mixture of anti‐GNAT3 and blocking peptide (1:50) in blocking buffer. Then, after the cryosections were washed in PBS (3 × 10 min), they were incubated for 1 h in secondary antibodies: Alexa633 or Alexa488 goat anti‐rabbit (1:400, Molecular Probes, USA). After the slides were washed in PBS (3 × 10 min), they were covered with an antifluorescence quenching agent. Images were obtained using a confocal microscope (Leica TCS SP5, Leica Microsystems Inc., Mannheim, Germany).

### 2.7 Two‐bottle preference tests

The two‐bottle preference test was performed as described previously.^37^ Taste preferences were studied using a 48‐h two‐bottle preference test with the following taste compounds: sucrose (Figure 4A–C), monosodium glutamate (Figure 4A–C), citric acid (Figure 4A–C), CYX (Figures 4 and 5), NaCl (Figure 4A–C), quinine (Figure 5D–F), CuB (Figure 6C,D), and DB (Figure 6A,B). The concentration of the test compounds is shown in the figure. Taste solutions were prepared fresh and stored in a glass bottle until further analysis; 12–13 C57BL/6 wild‐type (WT) and mutant mice in each group (12 *Tas2r104* ^*−/−*^ */Tas2r105* ^*−/−*^, 13 *Tas2r105* ^*−/−*^ */Tas2r114* ^*−/−*^, 13 *Tas2r104* ^*−/−*^ */Tas2r105* ^*−/−*^ */Tas2r114* ^*−/−*^, 12 *Gnat3* ^*−/−*^, 12 *Gnat3* ^*−/−*^ *‐Tas2r104* ^*−/−*^ */Tas2r105* ^*−/−*^) were involved in the following experiments. The mice, sex in half, were aged between 60 and 120 days at the beginning of the experiment and had similar body weight (WT = 19.1 ± 0.4 g, KO = 18.8 ± 0.5 g), which was weighed at the start of the first experiment.

Mice were randomly divided into individual cages in which two 100‐mL test bottles were placed. In each experiment, the mice were assigned two 100‐mL test bottles containing distilled water for 48 h, and they chose between distilled water and various concentrations of taste compounds shown in Figures 4, 5, 6. Each compound was tested for 48 h. During the 48‐h test, the places of the two test bottles were switched every 24 h. The two test bottles were weighed at the beginning and at the end of each 48‐h test; intakes were obtained by subtracting the end weight from the start weight. Preference scores were obtained by the intake of the solution divided by the total intake and expressed as a percentage.

### 2.8 Statistical analyses

All data are presented as mean ± standard error. We analyzed the differences among experimental groups using SPSS 21.0; two‐way analysis of variance was used to compare multigroup variables, and Student's *t*‐test was used to compare two independent groups. Statistical results are provided in the figure legends for each data set. Statistical significance was set at *p* < 0.05.

## 3 RESULTS

### 3.1 Precise deletion of bitter taste receptors using multiple sgRNAs and heritability of dual bitter taste receptor mutation

**FIGURE 1:**
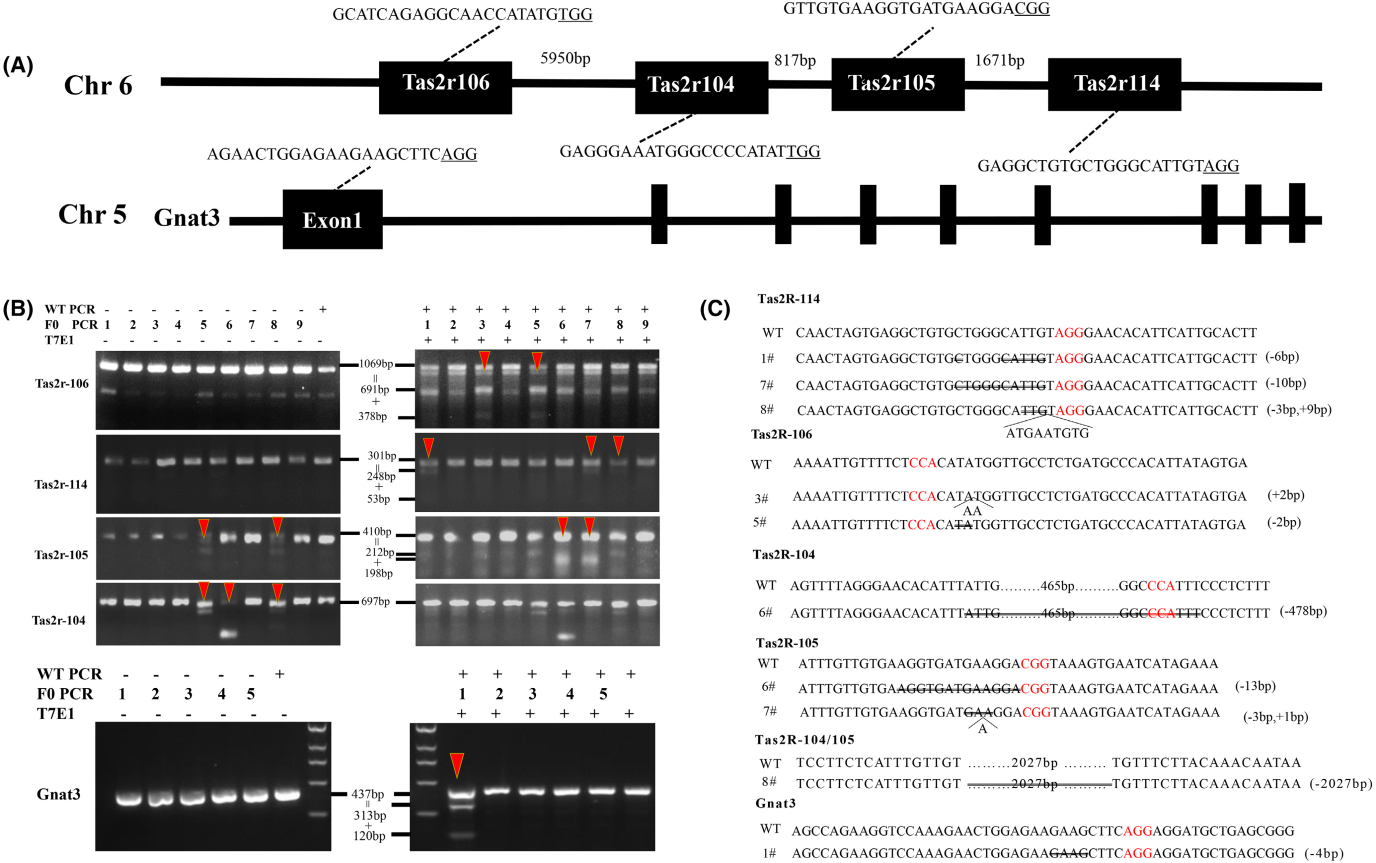
Genetic mutations of *Tas2rs* and *Gnat3* mediated by sgRNA/Cas9 mRNA mixtures. (A) Target sites of the four sgRNA at the *Tas2r106/Tas2r104/Tas2r105/Tas2r114* gene cluster and one sgRNA at the *Gnat3* gene locus. Black rectangles represent exons of *Tas2rs and Gnat3* genes. Target sites of SgRNA are shown by sequences. Protospacer adjacent motif (PAM) sites are underlined. (B) Representative electrophoretogram of F0 mice genotyping. Genome modifications were screened using PCR (polymerase chain reaction) and T7EN1 assays at the target sites in these founders. The number in the center indicates the size of PCR and digestion products; the red triangle indicates the occurrence of nucleotide deletion. (C) Sequencing of mutant alleles present in the F0 founders. Red color indicates the PAM sites; =, deletion; ^, insertion; WT, wild‐type control.

Most of the *Tas2rs* genes are located on the same chromosome in separate clusters defined by their physical locations. It is difficult to produce multiple *Tas2rs* gene KO using conventional gene targeting technology. Recent studies have shown that CRISPR/Cas9 technology is an efficient genome editing system. Therefore, we tried to use CRISPR/Cas9 system to produce genetic mutations in the *Tas2rs* gene cluster, including *Tas2r106*, *Tas2r104*, *Tas2r105*, and *Tas2r114*. One sgRNA was designed for each *Tas2rs* (Figure 1A). A mixture consisting of Cas9 mRNA and four sgRNA mRNAs was microinjected into the cytoplasm of the zygotes. Sixty‐eight embryos were injected, and nine mice were born from the 60 embryos transferred (i.e., 15% livebirth rate). PCR and T7EN1 assays were used to screen genetic mutation at the target sites. The PCR results indicated that six of the nine founders (66.7%) harbored genetic mutation in at least one targeted gene (Figure 1B). Two of them (6 and 7) were males, and the other four (1, 3, 5, and 8) were females. Amplification products were cloned and sequenced for further confirmation (Figure 1C). To study whether the mutation was heritable, four founders (3, 6, 7, and 8) were crossed with C57BL/6 mice. Genotyping results of F2 animals showed that all founders (3, 6, 7, and 8) passed the mutation to their offspring and produced four lines of *Tas2rs‐*modified mice (Figure S1). Of these, one line was triple edited (8), and two lines were double edited (6 and 7). Our results also revealed that founder 8 was genetically mosaic (Figure 1B; Figure S1J), which was consistent with previous reports that most of the mutants that develop from the injected or electroporated zygotes were genetically mosaic.^28^, ^38^

Sequencing results revealed that the triple‐edited line (8) had a 9‐bp insertion in *Tas2r114* locus and a large deletion (2027 bp) between the target sites of *Tas2r104* sgRNA and *Tas2r105* sgRNA (Figure 1C). Thus, both TAS2R104 and TAS2R105 protein sequences were disrupted in this triple‐edited mice. But a large deletion may generate a fusion gene, which contained 447 bp of *Tas2r105* and 156 bp of *Tas2r104*, and intact *5′UTR* of *Tas2r105* gene and *3′UTR* of *Tas2r104* gene. The 9‐bp insertion in *Tas2r114* leads to a short in‐frame shift, so TAS2R114 may still be functional in this line.

The double‐edited line (6) showed a large fragment deletion of 478 bp in *Tas2r104* around its sgRNA location and a 13‐bp deletion in *Tas2r105* around the sgRNA location. The 13‐bp deletion in *Tas2r105* creates a frameshift, consequently disrupting its function. A 478‐bp deletion causes a 157‐amino acid deletion in TAS2R104, which might result in a frameshift in the protein sequence. In the second double‐edited line (7), both the 10‐bp deletion in *Tas2r114* and the 3‐bp deletion, 1‐bp insertion in *Tas2r105* causes a frameshift, consequently disrupting their function. In the third line, a 2‐bp insertion may cause a frameshift in *Tas2r106* (Figure 1C).

To block bitter taste signal pathway, we also developed *Gnat3* mutant mice using CRISPR/Cas9 technology. One specific sgRNA for *Gnat3* gene was designed. *Gnat3* founder mice were obtained using the aforementioned procedure. A 4‐bp deletion was confirmed by T7EN1 assays and sequencing (Figure 1C). Then, founder mice were crossed with C57BL/6 mice, passed the mutation to their offspring, and gave rise to *Gnat3* mutant mice. After at least six rounds of upgrading with C57BL/6 mice, *Gnat3* ^+/−^ heterozygote mice were intercrossed to obtain homozygote mice. *Gnat3* ^−/−^ mice were crossed with *Tas2r104* ^*−/−*^ */Tas2r105* ^*−/−*^ mice. After several rounds of crossbreeding, we finally obtained double‐KO *Gnat3* ^−/−^‐Tas2r104^−/−^/*Tas2r105* ^−/−^ mice.

### 3.2 Mutation of bitter taste receptors did not affect the expression of *Plcβ2* and *Gnat3* in taste buds

**FIGURE 2:**
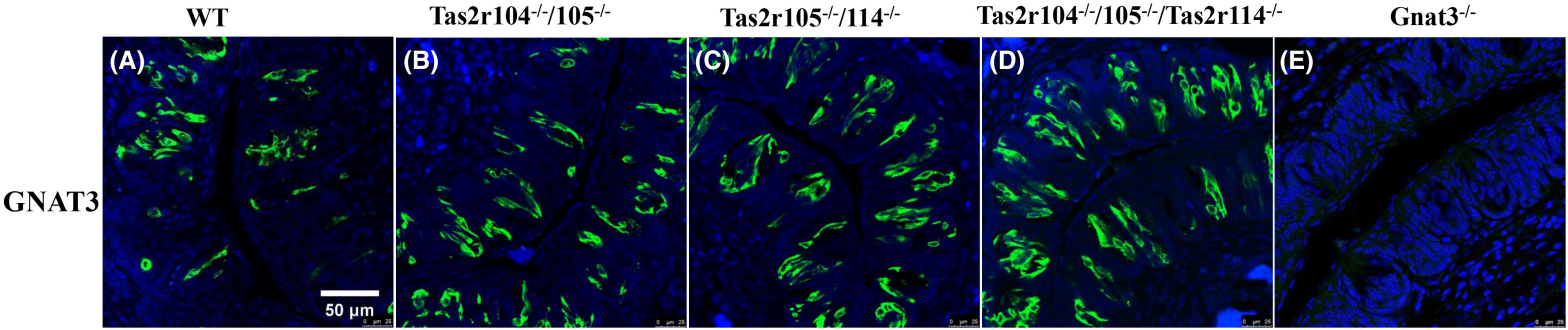
GNAT3 expression in the *Tas2rs* and *Gnat3* mutant mice. Microscopic images of frozen sections of taste bud–containing sections from circumvallate papillae from (A) WT (wild‐type) and (B–E) mutant mice obtained using indirect immunofluorescence with specific antibodies directed against GNAT3. (A) WT, (B) *Tas2r104* ^*−/−*^/*Tas2r105* ^*−/−*^ mice, (C) *Tas2r105* ^*−/−*^/*Tas2r114* ^*−/−*^ mice, (D) *Tas2r104* ^*−/−*^/*Tas2r105* ^*−/−*^/*Tas2r114* ^*−/−*^ mice, and (E) Gnat3^*−/−*^ mice. Scale bar: 50 μm.

**FIGURE 3:**
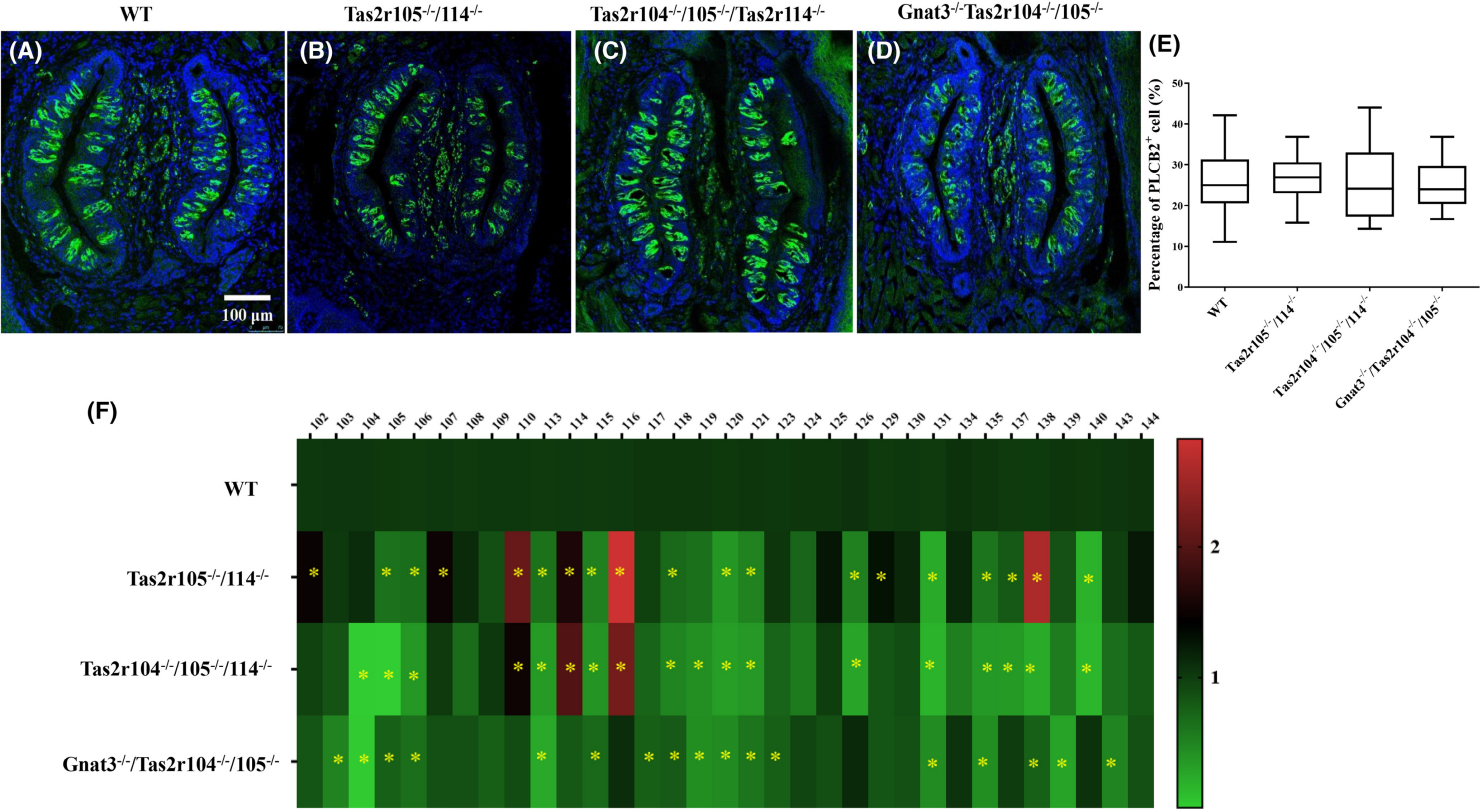
Genetic mutation of *mTas2rs* has no effect on the PLCβ2‐positive cell number in taste buds but can affect the expression profile of *mTas2rs* family. (A–D) Representative image of immunofluorescence staining with anti‐PLCβ2 in taste buds. Scale bar: 100 μm. (E) PLCβ2‐positive cell count in taste buds from these mutant mice. (F) The changed expression profile of *mTas2r* family revealed using qRT‐PCR (quantitative real‐time polymerase chain reaction) analysis. Statistical significance of differences between WT (wild‐type) and *Tas2rs* mutant mice was assessed using Student's *t*‐test, *p < 0.05.

After successfully developing several *Tas2rs* KO and double‐KO mouse models, including *Tas2r104* ^*−/−*^/*Tas2r105* ^*−/−*^, *Tas2r105* ^*−/−*^/*Tas2r114* ^*−/*−^, *Tas2r104* ^*−/−*^/*Tas2r105* ^*−/−*^/*Tas2r114* ^*−/−*^, *Tas2r106* ^*−/−*^, and *Gnat3* ^−/−^ *‐Tas2r104* ^*−/−*^ */Tas2r105* ^*−/−*^, we hoped to further investigate the effect of genetic mutation on taste‐related gene expression. Taste GPCRs specific for sweet or bitter are expressed in any given type II cell separately.^1^ It is well known that representative molecular markers for type II taste cells are *Gnat3* and *Plcβ2*.^39^ First, we measured the expression of *Gnat3* and *Plcβ2* in taste buds from these mutant mice using indirect fluorescent antibody assay. Immunostaining with GNAT3 antibody showed that the expression of GNAT3 (Figure 2A–D) in taste buds from *mTas2rs* mutant mice was similar to that of WT mice. But the expression of GNAT3 (Figure 2E) was deleted in taste buds from *Gnat3* ^−/−^ mutant mice, indicating that a 4‐bp deletion can cause a frameshift in *Gnat3* gene. We observed a similar expression pattern of PLCβ2 in taste buds from *Tas2rs* mutant and *Gnat3* ^−/*−*^ *‐Tas2r104* ^*−/−*^ */Tas2r105* ^−/−^ mice with that of WT mice (Figure 3A–D). PLCβ2‐positive cells were also counted in taste buds in mutant and WT mice, but no significant difference was observed among them (Figure 3E).

Then, we employed qRT–PCR to study whether the genetic mutation of *Tas2r105* cluster affects the expression level of other *Tas2rs* genes. The expression level of 19 *mTas2rs* in *Tas2r105* ^*−/−*^/*Tas2r114* ^*−/*−^ mice was significantly different from that in WT mice (Figure 3F). In *Tas2r104* ^*−/−*^/*Tas2r105* ^*−/−*^/*Tas2r114* ^*−/−*^ mice, the expression level of 18 *mTas2rs* was significantly changed (Figure 3F); 17 *mTas2rs* in *Gnat3* ^−/−^‐Tas2r104^−/−^/*Tas2r105* ^−/−^ mice were found to be significantly different compared to those in WT mice. In three mutant mice, several *mTas2rs*, including *mTas106*, *mTas120*, *mTas*121, *mTas131*, and *mTas135*, were significantly downregulated. *mTas126* and *mTas137* were significantly downregulated in *Tas2r105* ^*−/−*^/*Tas2r114* ^*−/*−^ and *Tas2r104* ^*−/−*^/*Tas2r105* ^*−/−*^/*Tas2r114* ^*−/−*^ mice. But the expression level of *Tas2r114* and *Tas2r110* was significantly upregulated in two mutant mice. In addition, we observed the expression of *Tas2r104/105* fusion gene, produced by a 2027‐bp deletion between the target sites of *Tas2r104* sgRNA and *Tas2r105* sgRNA.

### 3.3 Two‐bottle preference tests: Five basic tastes

**FIGURE 4:**
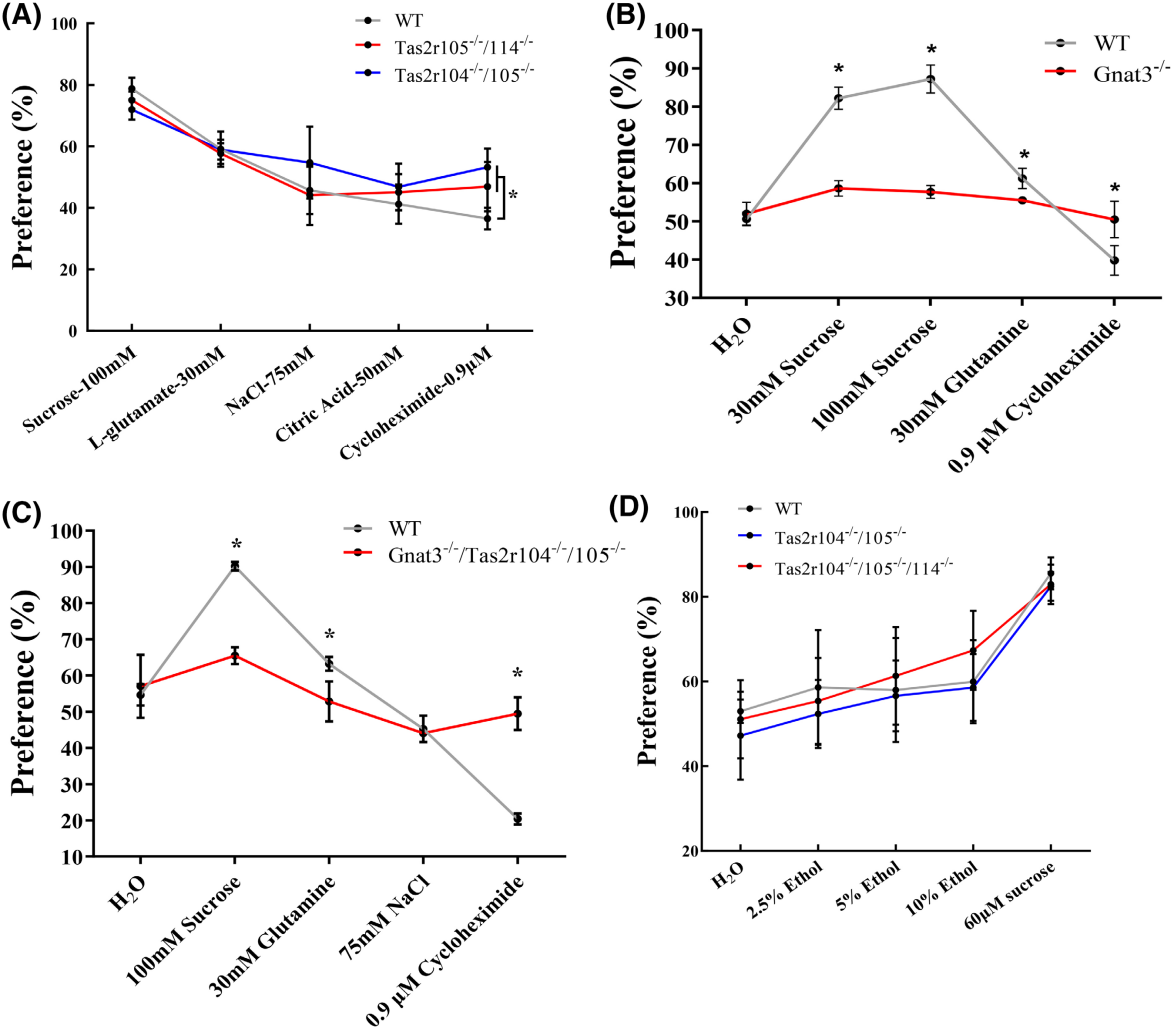
Results of two‐bottle preference tests in wild‐type and mutant mice. (A) Mean preference ratios from 48‐h two‐bottle preference tests (tastant vs. water), comparing responses of mutant mice (*Tas2r104* ^*−/−*^/*Tas2r105* ^*−/−*^, *Tas2r105* ^*−/−*^/*Tas2r114* ^*−/−*^) versus WT (wild‐type) controls to 100 mM sucrose, 30 mM l‐glutamate, 75 mM NaCl, 50 mM citric acid, and 0.9 μM cycloheximide. For each group, *n* = 12. (B) Mean preference ratios from 48‐h two‐bottle preference tests (tastant vs. water), comparing responses of mutant mice (*Gnat3* ^*−/−*^) versus WT controls to 30 mM sucrose, 100 mM sucrose, 30 mM l‐glutamate, and 0.9 μM cycloheximide. For each group, *n* = 12. (C) Mean preference ratios from 48‐h two‐bottle preference tests (tastant vs. water), comparing responses of mutant mice (*Gnat3* ^*−/−*^‐*Tas2r104* ^*−/−*^/*Tas2r105* ^*−/−*^) versus WT controls to 100 mM sucrose, 30 mM l‐glutamate, 75 mM NaCl, and 0.9 μM cycloheximide. For each group, *n* = 12. (D) Mean preference ratios from 48‐h two‐bottle preference tests (tastant vs. water), comparing responses of mutant mice (*Tas2r104* ^*−/−*^/*Tas2r105* ^*−/−*^, *Tas2r104* ^*−/−*^/*Tas2r105* ^*−/−*^/*Tas2r114* ^*−/−*^) versus WT controls to 2.5%, 5%, 10%, 60 mM sucrose. For each group, *n* = 13. Statistical significance of differences between WT and *Tas2rs* mice were assessed with Student’s *t*‐test, *p < 0.05.

Behavioral tests were used to investigate the responses of mutant mice (*Tas2r104* ^*−/−*^/*Tas2r105* ^*−/−*^, *Tas2r105* ^*−/−*^/*Tas2r114* ^*−/−*^) to taste compounds representing five taste qualities (sweet, bitter, salty, sour, and umami). In two‐bottle preference tests, the mutant mice showed indifference to 100 mM sucrose, 75 mM NaCl, 50 mM citric acid, and 30 mM l‐glutamate, similar to the preference in WT littermate controls. It has been reported that *Tas2r105* is the principal bitter taste receptor of CYX.^40^ Thus, the genetic mutation of *Tas2r105* gene should eliminate most responses to CYX. As expected, Figure 4A showed that the mutant mice (*Tas2r104* ^*−/−*^/*Tas2r105* ^*−/−*^, *Tas2r105* ^*−/−*^/*Tas2r114* ^*−/−*^) had a remarkable and selective loss of responses to 0.9 μM CYX. We also investigated the responses of mutant mice (*Gnat3* ^*−/−*^ and *Gnat3* ^*−/−*^ *‐Tas2r104* ^*−/−*^/*Tas2r105* ^*−/−*^) to taste compounds. *Gnat3* ^*−/−*^ mutant mice lost perception not only to low (30 mM) and high (100 mM) concentration sucrose but also to 30 mM glutamine and 0.9 μM CYX (Figure 4B). A similar phenotype was also observed in *Gnat3* ^*−/−*^ *‐Tas2r104* ^*−/−*^/*Tas2r105* ^*−/−*^ double mutant mice (Figure 4C).

Previous studies have revealed that ethanol may be involved in the perception of oral adaptation to bitter‐tasting stimuli.^41^, ^42^ To further study the taste perception of the *Tas2rs* mutant mice, different concentrations of ethanol were tested using two‐bottle preference tests. WT mice showed the expected preference for all concentrations of ethanol. Like these controls, the double‐ (*Tas2r104* ^*−/−*^/*Tas2r105* ^*−/*^) and triple‐ (*Tas2r104* ^*−/−*^/*Tas2r105* ^*−/−*^/*Tas2r114* ^*−−*^) mutant mice also preferred any ethanol concentration (Figure 4D).

### 3.4 Two‐bottle preference tests: CYX concentration series

**FIGURE 5:**
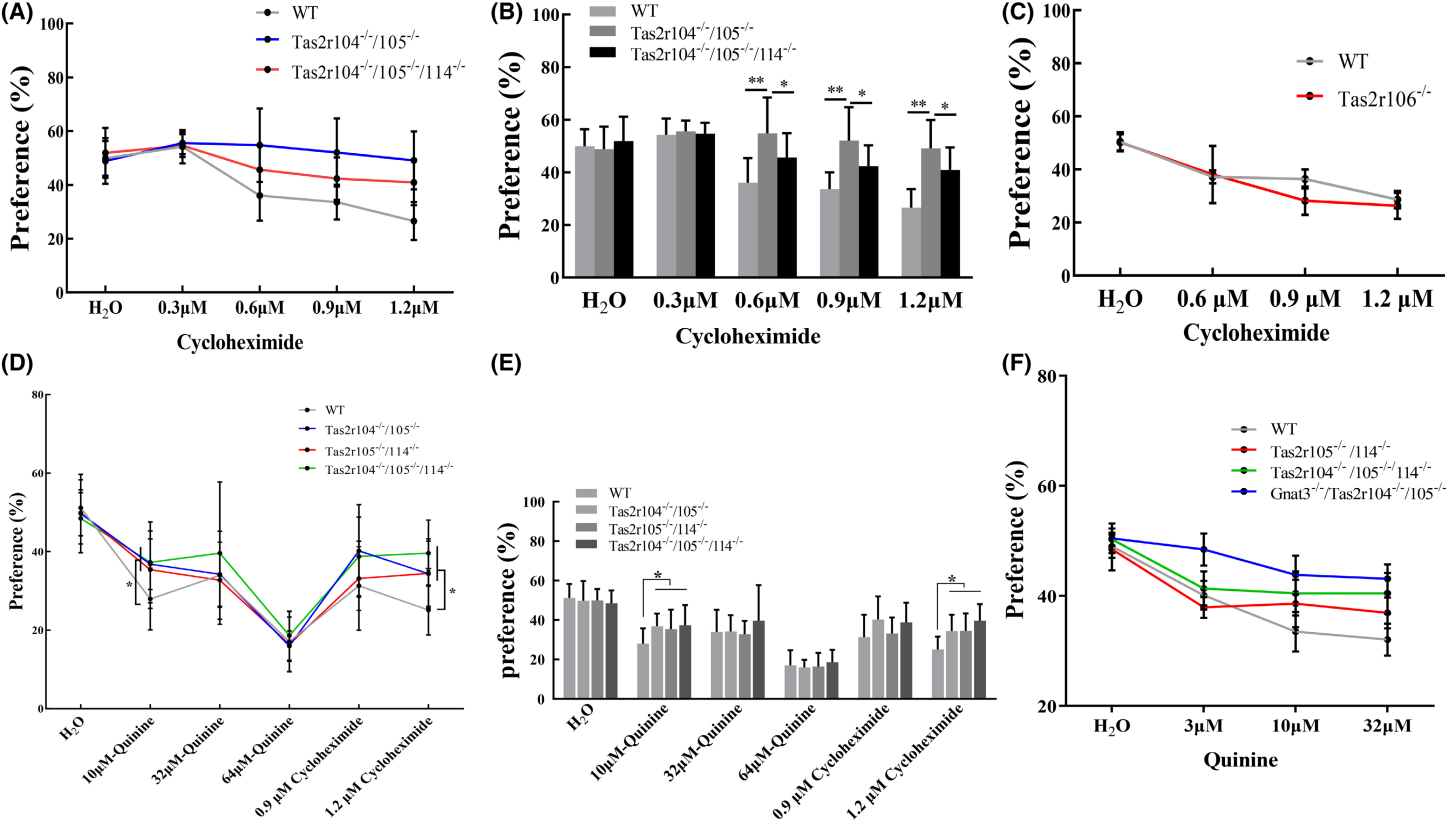
Mutant mice show a strong and selective impairment in their ability to taste cycloheximide. (A, B) Mean preference ratios from 48‐h two‐bottle preference tests (tastant vs. water), comparing responses of mutant mice (*Tas2r104* ^*−/−*^ */Tas2r105* ^*−/−*^, *Tas2r104* ^*−/−*^ */Tas2r105* ^*−/−*^ */Tas2r114* ^*−/−*^) versus WT (wild‐type) controls to 0.3, 0.6, 0.9, and 1.2 μM cycloheximide. For each group, *n* = 13. (C) Mean preference ratios from 48‐h two‐bottle preference tests (tastant vs. water), comparing responses of mutant mice (*Tas2r106* ^*−/−*^) versus WT controls to 0.6, 0.9, and 1.2 μM cycloheximide. For each group, *n* = 12. (D, E) Mean preference ratios from 48‐h two‐bottle preference tests (tastant vs. water), comparing responses of mutant mice (*Tas2r104* ^*−/−*^/*Tas2r105* ^*−/−*^, *Tas2r105* ^*−/−*^/*Tas2r114* ^*−/−*^, *Tas2r104* ^*−/−*^/*Tas2r105* ^*−/−*^/*Tas2r114* ^*−/−*^) versus WT controls to 10, 32, and 64 μM quinine dihydrochloride (QHCL) and 0.9 and 1.2 μM cycloheximide. For each group, *n* = 13. (F) Mean preference ratios from 48‐h two‐bottle preference tests (tastant vs. water), comparing responses of mutant mice (*Tas2r105* ^*−/−*^/*Tas2r114* ^*−/−*^, *Tas2r104* ^*−/−*^/*Tas2r105* ^*−/−*^/*Tas2r114* ^*−/−*^ and *Gnat3* ^*−/−*^ *‐Tas2r104* ^*−/−*^ */Tas2r105* ^*−/−*^) versus WT controls to 3, 10, and 32 μM QHCL. For each group, *n* = 13. Statistical significance of differences between WT and *Tas2rs* mice were assessed with Student’s *t*‐test, *p < 0.05.

To further study the threshold sensitivity of the mutant mice (*Tas2r104* ^*−/−*^/*Tas2r105* ^*−/−*^, *Tas2r104* ^*−/−*^/*Tas2r105* ^*−/−*^/*Tas2r114* ^*−/−*^, *Tas2r106* ^*−/−*^) for CYX, we tested these mice with different concentrations of CYX; 0.6, 0.9, and 1.2 μM CYX were preferred by these mice with the genetic mutations of *Tas2r105* (*Tas2r104* ^*−/−*^/*Tas2r105* ^*−/−*^, *Tas2r104* ^*−/−*^/*Tas2r105* ^*−/−*^/*Tas2r114* ^*−/−*^) (Figure 5A). Interestingly, the *Tas2r104* ^*−/−*^/*Tas2r105* ^*−/−*^ mice had a preference score similar to 50% (indifference) for 1.2 μM CYX. *Tas2r104* ^*−/−*^/*Tas2r105* ^*−/−*^/*Tas2r114* ^*−/−*^ mice showed a preference score between the *Tas2r104* ^*−/−*^/*Tas2r105* ^*−/−*^ mice and WT mice (Figure 5B). Figure 5C shows that 0.6, 0.9, and 1.2 μM CYX were not preferred by WT groups and *Tas2r106* ^*−/−*^ mice equally, indicating that *Tas2r106* may not be involved in the perception of CYX.

To further investigate the response of other bitter tastants, we tested these mice with different concentrations of quinine (QHCI). The mutant and the WT mice strongly avoided QHCI, but the preference score in the mutant mice was higher than that in the WT mice for 10 μM QHCI (Figure 5D,E), indicating that their sensitivity was greatly decreased compared to WT mice in this concentration. Subsequently, the mutant and the WT mice strongly avoided CYX, but the preference score in the mutant mice was higher than that in the WT mice for 1.2 μM CYX (Figure 5D,E). This indicated that QHCI pre‐intake may increase the threshold sensitivity of bitter tastants, but perception for CYX still decreased in these mutant mice. These mutant mice of *Tas2rs* gene can still detect and avoid high concentrations of CYX.

### 3.5 Two‐bottle preference tests: QHCI, DB, and CuB

**FIGURE 6:**
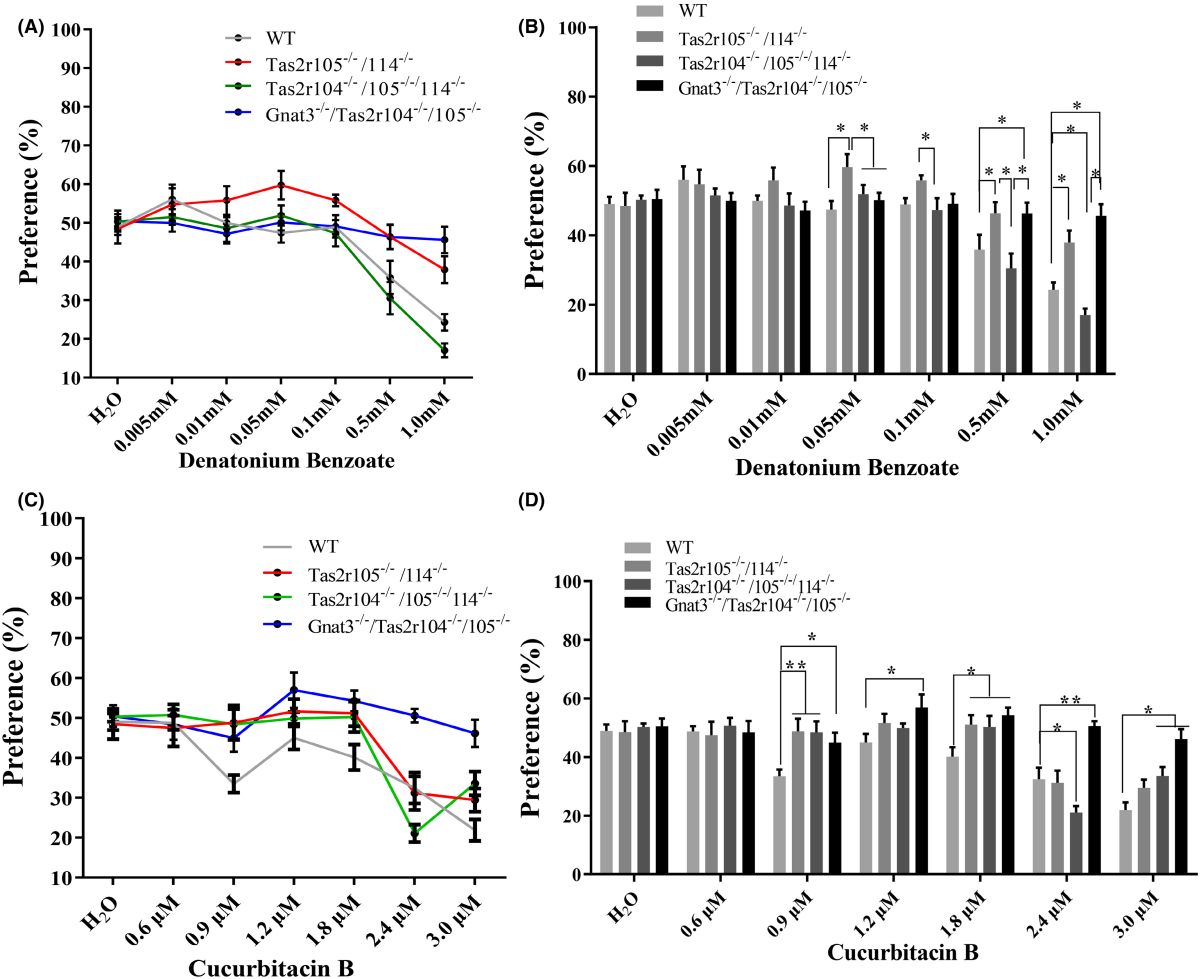
Two‐bottle test preference scores of WT (wild‐type) and mutant mice given an ascending concentration series of denatonium benzoate (DB) and cucurbitacin B (CuB). (A, B) Mean preference ratios from 48‐h two‐bottle preference tests (tastant vs. water), comparing responses of mutant mice (*Tas2r105* ^*−/−*^/*Tas2r114* ^*−/−*^, *Tas2r104* ^*−/−*^/*Tas2r105* ^*−/−*^/*Tas2r114* ^*−/−*^, and *Gnat3* ^*−/−*^ *‐Tas2r104* ^*−/−*^/*Tas2r105* ^*−/−*^) versus WT controls to 0.005, 0.01, 0.05, 0.1, 0.5, and 1.0 μM DB. For each group, *n* = 13. (C, D) Mean preference ratios from 48‐h two‐bottle preference tests (tastant vs. water), comparing responses of mutant mice (*Tas2r105* ^*−/−*^/*Tas2r114* ^*−/−*^, *Tas2r104* ^*−/−*^/*Tas2r105* ^*−/−*^/*Tas2r114* ^*−/−*^, and *Gnat3* ^*−/−*^ *‐Tas2r104* ^*−/−*^/*Tas2r105* ^*−/−*^) versus WT controls to 0.6, 0.9, 1.2, 1.8, 2.4, and 3.0 μM CuB. For each group, *n* = 13. Asterisks indicate statistically significant differences between the responses of two groups (*p* < 0.05).

As the *Tas2rs* mutant mice unexpectedly showed preferences for 10 mM quinine, we then considered if they altered response to general bitter sensory experience. Consequently, *Tas2rs* mutant mice, double mutant mice, and WT mice were tested with QHCI and DB. DB and QHCI have structurally dissimilar stimuli, and they stimulate different subsets of rat taste receptor cells (TRCs).^43^, ^44^ The preference ratios for 10 μM QHCI were significantly higher for the mutant mice than for the WT mice (Figure 5F). DB solutions of varying concentrations were presented to mutant and WT mice to test for genotype differences in preference ratios (Figure 6A). The analyses of these data showed that *Tas2r105* ^*−/−*^/*Tas2r114* ^*−/−*^ and *Gnat3* ^*−/−*^ *‐Tas2r104* ^*−/−*^/*Tas2r105* ^*−/−*^ mice had significantly higher preference scores for 0.5 and 1 mM DB solution than *Tas2r104* ^*−/−*^/*Tas2r105* ^*−/−*^/*Tas2r114* ^*−/−*^ and WT mice (Figure 6B).

In a heterologous cell expression system, CuB had been identified as cognate agonists for mTAS2R105 and mTAS2R114.^25^ We hypothesized that if two taste receptors contribute to CuB preference, the mutant mice (*Tas2r104* ^*−/−*^/*Tas2r105* ^*−/−*^ and *Tas2r104* ^*−/−*^/*Tas2r105* ^*−/−*^/*Tas2r114* ^*−/−*^) would not detect CuB and would show preference ratios similar to that of water. These mutant mice were given various concentrations of CuB, and their preference ratios were analyzed. The analysis of the CuB intake data indicated that the preference ratios for the mutant mice were significantly larger at 0.9 and 1.8 μM CuB relative to those of the WT mice (Figure 6C,D). The double mutant mice (*Gnat3* ^*−/−*^‐*Tas2r104* ^*−/−*^/*Tas2r105* ^*−/−*^) still failed to discriminate higher concentrations of CuB (2.4 and 3.0 μM) from water (Figure 6C,D). The current results suggest that the mutation of *Tas2r105* and *Tas2r114* indeed attenuated the taste perception to CuB and bitter taste signal pathway contributes to CuB perception.

## 4 DISCUSSION

A previous study has shown that muroid cluster I contains *Tas2r107*, *Tas2r106*, *Tas2r105*, *Tas2r114*, and *Tas2r104*, which have a common ancestor with human TAS2R10.^18^ Genetic deletion of *Tas2r105* eliminates more neural responses and behavioral aversion to CYX. But taste perception for denatonium, 6‐n‐propyl thiouracil, and quinine was not changed in *Tas2r105* null mice.^40^ Recently, bitter taste signaling pathway is found in a large number of tissues and organs, and is intensively involved in biological functions in extraoral tissues.^2^, ^5^, ^11^, ^15^, ^22^, ^45^, ^46^, ^47^, ^48^ However, the TAS2Rs biological function in extra lingual tissue still needs to be further studied. Due to the expression of multiple *Tas2r* genes in a given extraoral tissue, a better way is to develop and use multiple *Tas2r*s KO mice. Because there are 35 *Tas2r* genes in mice, it would be an arduous task to produce multiple *Tas2rs* KO mice. However, the emergence of the CRISPR/Cas9 technology enables us to efficiently develop multiple *Tas2rs* KO mice. In this study, several multiple *Tas2rs* KO mice, including *Tas2r106* ^−/−^, *Tas2r104* ^*−/−*^ */Tas2r105* ^*−*/−^, *Tas2r105* ^*−/−*^ */Tas2r114* ^−/−^, and *Tas2r104* ^*−/−*^/*Tas2r105* ^*−/−*^ */Tas2r114* ^−/−^, were developed using the CRISPR/Cas9 gene‐editing technique. Subsequent breeding work further showed that the genetic mutation in *Tas2r104/Tas2r105/Tas2r114* would be linked to transmit to the next generation. Another study also developed *Tas2r* triple (*Tas2r143*/*Tas2r135*/*Tas2r126*) KO mice using the CRISPR/Cas9 gene‐editing technique and successfully deleted the 27‐kb genomic fragment between two sgRNAs of *Tas2r135* and *Tas2r126*.^49^ Furthermore, it has been frequently reported that the CRISPR/Cas9 technique can produce large somatic and heritable deletions of genomic fragments.^50^ Therefore, the current success in developing multiple *Tas2rs* KO mice suggests that it is feasible for developing multiple *Tas2r* KO mice using the CRISPR/Cas9 technique.

Most of the murine *Tas2r* genes were distributed in eight loci.^17^, ^18^ Thus far, the regulation mechanism of *Tas2rs* gene expression remains unclear. Due to the altered expression of *Tas2r136* and *Tas2r137* in large airways in triple *Tas2r* KO mice (*Tas2r143*/*Tas2r135*/*Tas2r126*), the expression of *Tas2rs* may be regulated in a receptor type‐dependent manner in the airway.^49^ The *Tas2r104/Tas2r105/Tas2r114* genes are organized as a head‐to‐tail array within a single 6‐kb DNA fragment.^20^ A previous study has suggested that shared regulatory sequences may coordinately regulate the expression of *Tas2rs* gene cluster.^20^ In this study, a deletion of interval sequence (817 bp) between *Tas2r104* and *Tas2r105* genes indeed affected the expression of *mTas2rs* in taste buds in the triple mutant mice. Meanwhile, we also observed the changed expression of *Tas2rs* in taste buds from double mutant mice. This further contributed to a different change in bitter taste perception. It is worth noting that the *Tas2r104/Tas2r105/Tas2r114* genes are often coexpressed in many extraoral tissues with the characteristics of tissue‐specific expression.^7^, ^9^, ^24^, ^51^ Thus, the expression regulation of this gene cluster in extraoral tissues still needs to be further studied. We speculate that these mutant mice, especially *Tas2r104* ^*−/−*^ */Tas2r105* ^*−/−*^ */Tas2r114* ^*−−−*^ triple mutant mice, may have an altered expression profile of *Tas2rs* in extraoral tissues, which can trigger deeper physiological significance. *Tas2rs114* expression, for instance, is low in lingual epithelia but much higher in testis,^24^ gut, and kidney.^7^, ^21^ It has shown that progesterone can stimulate *Tas2r114* and *Tas2r110* in HEK293T cells stably expressing Ga16gust44 system,^25^ indicating that hormones could function as *Tas2r* activators. Thus, our *Tas2r* KO mice should be a valuable means to evaluate the role of *Tas2r106/Tas2r104/Tas2r105/Tas2r114* gene cluster and allow us to check whether these TAS2Rs are really involved in the physiological functions in extraoral tissues and organs, such as lung, testis, gut, and kidney. Further studies may be critical for comprehending the role of these TAS2Rs in health and diseases.

We further measured the taste choices for these genetic mutants using two‐bottle preference assays. As expected, the genetic mutation/deletion of *Tas2r105* indeed eliminated the taste response to CYX but not to alcohol, sweet, umami, salty, and sour tastants. A further study also found that the genetic mutation of *Tas2r106* did not show a selective impairment in their ability to taste CYX. The heterologous expression analysis using the Ga16gust44 cell system also failed to identify any agnoists for *Tas2r106*.^25^ In this study, a 13‐ or 2‐bp deletion in *Tas2r105* and a 4‐bp deletion in *Gnat3* can cause a frameshift, adequately destroying its function. Thus, a 2‐bp insertion in *Tas2r106* was more likely to disrupt its function, indicating that *Tas2r106* may not be involved in the taste perception for CYX.

Another finding is that these *Tas2rs* mutant mice showed not only a changed perception ability for 10 μM QHCI and DB (0.5 and 1.0 mM) but also a reduced perception ability for CuB (0.9 and 1.8 μM). *Tas2r105* ^*−/−*^ */Tas2r114* ^*−/−*^ double mutant mice can discriminate 0.5 mM DB, but *Tas2r104* ^*−/−*^ */Tas2r105* ^*−/−*^ */Tas2r114* ^*−/−*^ triple mutant mice cannot. This indicated that the genetic mutation of three bitter receptors can result in a loss of sensitivity for 0.5 mM DB. qRT‐PCR results further showed that the deletion of interval sequence between *mTas2r104* and *mTas2r105* can alter the expression level of many *mTas2rs* genes in taste buds, for instance, downregulating the expression of *mTas2r135* and *mTas2r140*, which may contribute to loss of sensitivity for DB.^25^ In this study, two *Tas2rs* mutant mice showed an inconsistent taste perception to QHCI and DB, suggesting that two bitter substances can be sensed by a different set of receptors and/or a different subset of TRCs, which is consistent with previous reports.^43^, ^44^

CuB is one of the derivatives of cucurbitacins, including the triterpenoid structure, which serve significant bioactivities such as anti‐inflammatory effects, anticancer effects, antioxidative stress, and preventive effects against hepatotoxicity.^52^, ^53^ CuB can induce plasma GLP‐1 and insulin release in diabetic mice, which was involved in the activation of adenosine 5‘‐monophosphate (AMP)‐activated protein kinase through α‐gustducin and Gβγ‐signaling of taste receptors.^54^ Gustatory receptor is required for the taste sensation of CuB, which can activate Gr33a bitter‐sensing gustatory receptor neurons in *Drosophila melanogaster*.^55^ Using a heterologous HEK293T‐Gα16gust44 cell system, *Tas2r105* and *Tas2r114* were specifically activated by CuB.^25^ In this study, these mice, including the mutation of *mTas2r105* and *mTas2r11*4, were not able to sense 0.9 and 1.8 μM CuB. Furthermore, the *Gnat3* ^*−/−*^‐*Tas2r104* ^*−/−*^/*Tas2r105* ^*−/−*^ mice, due to the lack of GNAT3 and bitter taste receptors, did not respond to higher concentrations of CuB (2.4 and 3.0 μM). These results collectively demonstrate two points. First, *Tas2r105* and *Tas2r11*4 are indeed involved in the perception to CuB, but there may still be any unidentified chemoreceptor category to respond to CuB, because *Tas2r105* ^*−/−*^ */Tas2r114* ^*−/−*^ mutant mice can still respond to higher concentrations of CuB. Second, taste perception to CuB needs the *Gnat3*‐mediated receptor and its signaling pathway in mice.

Briefly, the CRISPR/Cas9 technique can effectively knock out multiple *Tas2rs* genes at once. Although genetic mutation of the these *Tas2rs* genes did not affect the expression of GNAT3 and PLCβ2, the expression profile of *Tas2rs* genes was changed in taste buds. Two‐bottle preference tests revealed that these mutant mice exhibited a decrease response to bitter tastants, including CYX, QHCI, DB, and CuB. These findings implied that these mutant mice would be a valuable means to understand the biological functions of TAS2Rs in extraoral tissues and investigate bitter compound–induced responses mediated by these TAS2Rs in many other extraoral tissues.

## AUTHOR CONTRIBUTIONS

Feng Li and Xiaohui Zhou conceived and designed the study; Bowen Niu completed the microinjection, produced the mutant mice, and performed quantitative PCR and immunostaining. Lingling Liu was involved in animal crossbreeding and behavior tests. Bowen Niu, Lingling Liu, and Qian Gao plotted the figures. Qian Gao, Meng‐Min Zhu, Lixiang Chen, Xiu‐Hua Peng, and Boying Qin performed the two‐bottle tests. Feng Li and Xiaohui Zhou drafted the manuscript; all authors revised the final version.

## FUNDING INFORMATION

This work was supported by the National Science and Technology Major Project (2017ZX10304402‐001‐006, 2017ZX10304402‐001‐012), Shanghai Science and Technology Commission “R&D Public Service Platform and Institutional Capacity Improvement Project” (21DZ2291300), and Start‐on Funding from Shanghai Public Health Clinical Center (KY‐GW‐2019‐11, KY‐GW‐2019‐19, and KY‐GW‐2021‐39).

## CONFLICT OF INTEREST STATEMENT

The authors declare that they have no conflicts of interest to this work. Xiaohui Zhou is an editorial board member of *AMEM* and a coauthor of this article. To minimize bias, he was excluded from all editorial decision making related to the acceptance of this article for publication.

## ETHICS STATEMENT

The study was approved by the Institute of Animal Use and Care Committee of Shanghai Public Health Clinical Center (GW2018‐A055‐01, GW2023‐A030‐01).

## Supporting information


**FIGURE S1** Screening of sgRNA/Cas9‐mediated on‐target cleavage of *Tas2rs* by the T7EN1 assay. (A–C) PCR (polymerase chain reaction) products from *Tas2rs*‐6 (*Tas2r104* ^*−/−*^/*Tas2r105* ^*−/−*^) homozygote, heterozygote, and wild‐type mice (WT) were subjected to the T7EN1 assay. The mutations were detected in (A) *Tas2r104* and (B) *Tas2r105* (B) but not in (C) *Tas2r114*. (D–F) PCR products from *Tas2rs*‐7 (*Tas2r105* ^*−/−*^/*Tas2r114* ^*−/−*^) homozygote, heterozygote, and WT mice were analyzed using the T7EN1 assay. The mutations were detected in (F) *Tas2r114* and (E) *Tas2r105* but not in (D) *Tas2r104* (D). G‐specific primers were used to amplify *Tas2r104* in *Tas2rs*‐8 mice (*Tas2r104* ^−/−^/*Tas2r105* ^*−/−*^/*Tas2r114* ^*−/−*^); a 697‐bp band was detected in PCR products from heterozygote but not in homozygote. H‐specific primers were used to amplify *Tas2r105* in *Tas2rs*‐8 mice; a 410‐bp band was detected in PCR product from heterozygote and wild type but not in homozygote. I PCR products from *Tas2rs*‐8 homozygote, heterozygote, and wild type mice were analyzed using the T7EN1 assay. The genetic mutations were found in *Tas2r114* gene. J Upstream primers from *Tas2r104* and downstream primers from *Tas2r105* were used to amplify genomic DNA. A 816‐bp band was found in *Tas2rs*‐8 homozygote mice. But a 2843 band was also found in heterozygote and wild type, indicating that a 2027‐bp size was deleted between *Tas2r104* and *Tas2r105* in *Tas2rs*‐8 homozygote.
