# Supplementary figures and images for: Genetic mutation of Tas2r104/Tas2r105/Tas2r114 cluster leads to a loss of taste perception to denatonium benzoate and cucurbitacin B
Source: Animal Model Exp Med. 2023 Dec 28;7(3):324–36. doi: 10.1002/ame2.12357 (PMC11228091; doi:10.1002/ame2.12357)

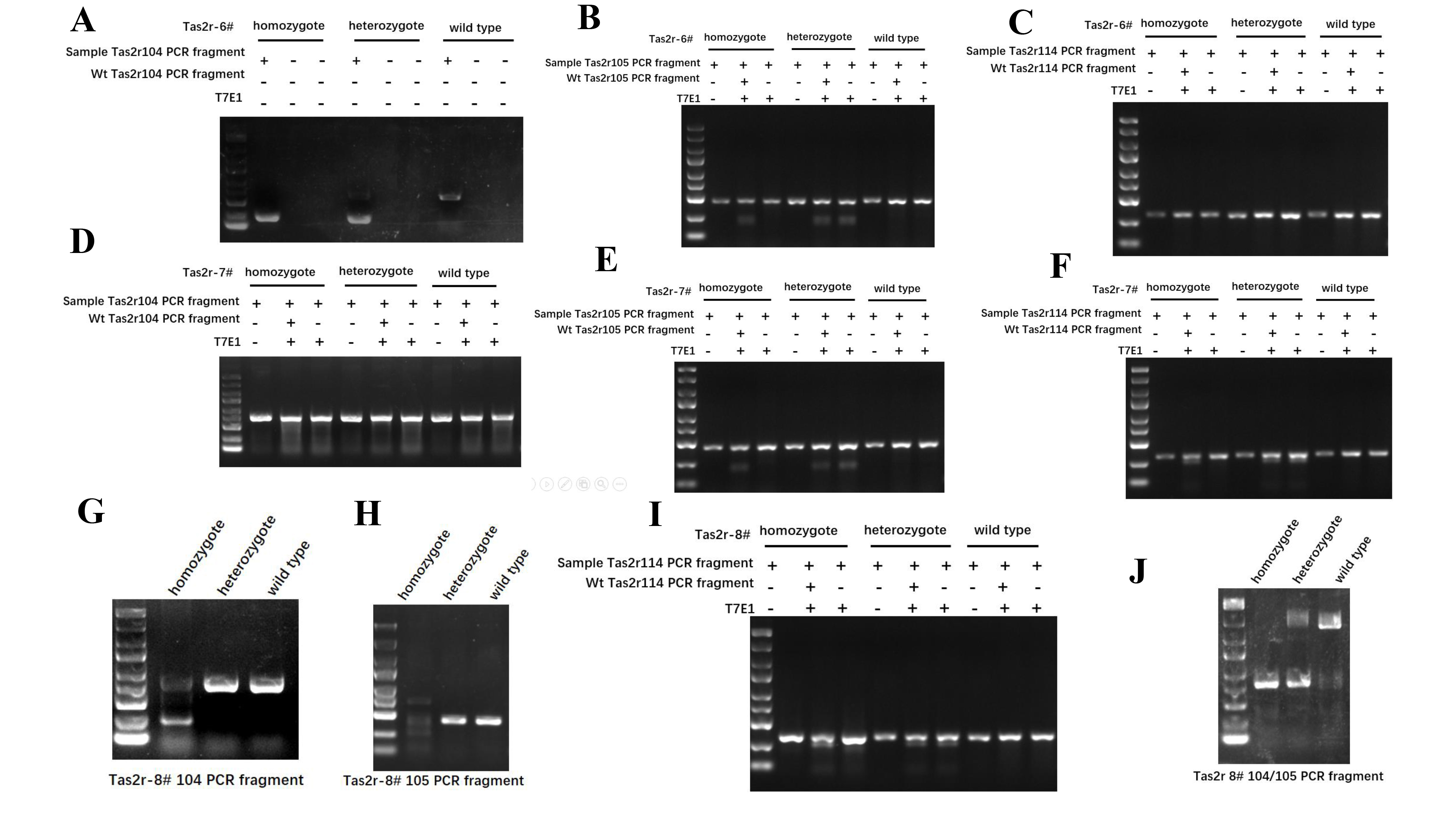

Supplement: Supplementary file 1 — FIGURE S1 Screening of sgRNA/Cas9‐mediated on‐target cleavage of Tas2rs by the T7EN1 assay. (A–C) PCR (polymerase chain reaction) products from Tas2rs‐6 (Tas2r104 −/− /Tas2r105 −/− ) homozygote, heterozygote, and wild‐type mice (WT) were subjected to the T7EN1 assay. The mutations were detected in (A) Tas2r104 and (B) Tas2r105 (B) but not in (C) Tas2r114. (D–F) PCR products from Tas2rs‐7 (Tas2r105 −/− /Tas2r114 −/− ) homozygote, heterozygote, and WT mice were analyzed using the T7EN1 assay. The mutations were detected in (F) Tas2r114 and (E) Tas2r105 but not in (D) Tas2r104 (D). G‐specific primers were used to amplify Tas2r104 in Tas2rs‐8 mice (Tas2r104 −/−/Tas2r105 −/− /Tas2r114 −/− ); a 697‐bp band was detected in PCR products from heterozygote but not in homozygote. H‐specific primers were used to amplify Tas2r105 in Tas2rs‐8 mice; a 410‐bp band was detected in PCR product from heterozygote and wild type but not in homozygote. I PCR products from Tas2rs‐8 homozygote, heterozygote, and wild type mice were analyzed using the T7EN1 assay. The genetic mutations were found in Tas2r114 gene. J Upstream primers from Tas2r104 and downstream primers from Tas2r105 were used to amplify genomic DNA. A 816‐bp band was found in Tas2rs‐8 homozygote mice. But a 2843 band was also found in heterozygote and wild type, indicating that a 2027‐bp size was deleted between Tas2r104 and Tas2r105 in Tas2rs‐8 homozygote. [file AME2-7-324-s001.jpg]
